# Does interspecies hybridization affect the host specificity of parasites in cyprinid fish?

**DOI:** 10.1186/1756-3305-6-95

**Published:** 2013-04-12

**Authors:** Andrea Šimková, Martina Dávidová, Ivo Papoušek, Lukáš Vetešník

**Affiliations:** 1 Department of Botany and Zoology, Faculty of Science, Masaryk University, Kotlářská 2, 611 37, Brno, Czech Republic; 2 Institute of Vertebrate Biology, Academy of Sciences of the Czech Republic, v.v.i., Květná 8, 603 65, Brno, Czech Republic; 3 Department of Biology and Wildlife Diseases, Faculty of Veterinary Hygiene and Ecology, University of Veterinary and Pharmaceutical Sciences Brno, Palackého tř. 1/3, 612 42, Brno, Czech Republic

**Keywords:** Cyprinid fish, Interspecies hybridization, Metazoan parasites, Monogenea, Host specificity

## Abstract

**Background:**

Host specificity varies among parasite species. Some parasites are strictly host-specific, others show a specificity for congeneric or non-congeneric phylogenetically related host species, whilst some others are non-specific (generalists). Two cyprinids, *Cyprinus carpio* and *Carassius gibelio*, plus their respective hybrids were investigated for metazoan parasites. The aim of this study was to analyze whether interspecies hybridization affects host specificity. The different degrees of host specificity within a phylogenetic framework were taken into consideration (i.e. strict specialist, intermediate specialist, and intermediate generalist).

**Methods:**

Fish were collected during harvesting the pond and identified using meristic traits and molecular markers. Metazoan parasite species were collected. Host specificity of parasites was determined using the following classification: strict specialist, intermediate specialist, intermediate generalist and generalist. Parasite species richness was compared between parental species and their hybrids. The effect of host species on abundance of parasites differing in host specificity was tested.

**Results:**

Hybrids harbored more different parasite species but their total parasite abundance was lower in comparison with parental species. Interspecies hybridization affected the host specificity of ecto- and endoparasites. Parasite species exhibiting different degrees of host specificity for *C. carpio* and *C. gibelio* were also present in hybrids. The abundance of strict specialists of *C. carpio* was significantly higher in parental species than in hybrids. Intermediate generalists parasitizing *C. carpio* and *C. gibelio* as two phylogenetically closely related host species preferentially infected *C. gibelio* when compared to *C. carpio*, based on prevalence and maximum intensity of infection. Hybrids were less infected by intermediate generalists when compared to *C. gibelio*.

**Conclusions:**

This finding does not support strict co-adaptation between host and parasite genotypes resulting in narrow host specificity, and showed that hybrid genotypes are susceptible to parasites exhibiting host specificity. The immune mechanisms specific to parental species might represent potential mechanisms explaining the low abundance of parasites in *C. gibelio* x *C. carpio* hybrids.

## Background

Host specificity is commonly defined by means of a simple classification into specialists or generalists. A specialist parasitizes a single host species, whilst a generalist parasitizes several host species [1]. However, because of the multifaceted nature of host specificity, this traditional classification applied in many studies can mask the differences in host specificity of the parasites that are associated to ecological importance, phylogenetic relatedness or geographical distribution of their host species [2]. Therefore, host specificity can be expressed at different scales (see [2,3]). Considering a phylogenetic framework, the semiquantitative classification of host specificity taking into account the phylogenetic relatedness of host species was applied for congeneric monogenean parasites by Desdevises *et al*. [4] and Šimková *et al*. [5]. A strict specialist (or species-specific parasite) is a parasite species infecting a single host species [1,5], whilst other specialists may infect phylogenetically closely related hosts such are congeneric hosts (termed as intermediate specialists by Desdevises *et al*. [4]) or some parasites (termed as intermediate generalists by Desdevises *et al*. [4]) may infect non-congeneric but phylogenetically closely related host species e.g. hosts forming a monophyletic group [5].

Up to now, only a few studies investigating whether or not host specificity is affected by interspecies hybridization, a common phenomenon in animals, have been performed [6,7]. These studies were limited to one parasite species, but in natural conditions the hosts are parasitized by various parasite species, and parasite communities on hosts are formed by both specialists and generalists. Concerning cyprinid fish, it is estimated that more than 30% are hybrids [8]. Currently, there is a lack of knowledge concerning host susceptibility to metazoan parasites in fish hybrids, and only two studies dealing with the host specificity of some metazoan parasites in hybridizing cyprinid fish have been performed [9,10].

The susceptibility or resistance of hybrids to viral, protozoan or helminth infection was investigated mainly in mice within their hybrid zones. It was shown that mouse hybrids are more susceptible to infection than their parental species due to genomic incompatibilities in the introgressed genomes of the hybrids [11-13]. However, mice trapped in the same part of the hybrid zone had two different phenotypes, susceptible and resistant, but an unfavorable genetic combination occurred more frequently in recombinant genotypes, producing a higher susceptibility to parasitic helminths [12]. The study of co-infection by two species, one protozoan and one helminth with different life traits, led to the suggestion that hybrid susceptibility is applied only to parasites that exert adequate constraints on their host to induce the selection of co-adapted genes of resistance [14].

Several studies aimed at investigating hybrid resistance to viral infection were performed in fish under experimental conditions, suggesting (conversely to mice) that fish hybrids are less susceptible than their parental species (for instance [15,16]). The experimental study of salmonid hybrids showed that the abundance of two *Gyrodactylus* species (Monogenea) was lower on hybrids than on their respective pure-bred natural hosts, and a parental sire-and-dam influence on the resistance of hybrids was observed [17]. The highly frequent natural hybrids of two cyprinid species, *Alburnus alburnus* and *Rutilus rubilio*, from Lake Mikri Prespa (Northern Greece) showed a high susceptibility to metazoan parasites when compared with pure species [9]. However, the hybrids of two cyprinids, native *Parachondrostoma toxostoma* and invasive *Chondrostoma nasus* in southern France, were less parasitized by ecto- and endoparasites than both pure species in the localities where these fish species live in sympatry [10].

Generally, ectoparasites with a direct life cycle are considered to be more specific than endoparasites with a complex life cycle. Among fish ectoparasites, Monogenea, which live on the gills, fins and skin, represent a parasite group with narrow host specificity. The genetically based host-specific barriers occurring due to co-evolution between a host and its specific parasites can determine the presence of specific parasites. Host resistance to two congeneric monogenean parasites, each of them specific to one parental species, was found in laboratory-raised F1 hybrids of anurans [7]. However, parasite infection in hybrid specimens is also determined by the degree of hybridization, i.e. the introgression rate, as was shown for gill *Diplozoon* species (Monogenea), in hybridizing the cyprinid fish species *Barbus barbus* and *B. meridionalis*[6].

Among cyprinid species, the hybrids between *Cyprinus carpio* and *Carassius gibelio* have previously been identified using morphological criteria and molecular markers (for instance [18]). However, reports of hybrids living in areas where pure cyprinid species live in sympatry are rare, particularly if the hybrids are identified using only morphological criteria (unpublished study). Concerning the parasitofauna of *C. carpio* and *C. gibelio*, both harbor several species specific or genus specific monogenean species.

The aim of this study was to investigate the metazoan parasite communities in *C. carpio* and *C. gibelio* and their respective hybrids. We focused on metazoan parasites exhibiting the different degrees of host specificity to analyze whether interspecies hybridization affects host specificity. The aspect of phylogenetic relatedness of host species was included in host specificity delimitation.

## Methods

For this study, 12 individuals of *C. carpio*, 14 individuals of *C. gibelio* and 13 individuals of their respective hybrids were investigated. All specimens were of the same age and were collected while harvesting the Hlohovecký fish pond (48°46'51"N, 16°47'2"E; Danube River Basin, the Czech Republic) in November. All hybrid specimens found during the harvesting were collected. The hybrids were identified using meristic traits such as number of gill rakers, length of upper and lower barbels, and number of scales in the lateral line. All investigated fish were fin-clipped and the clips were stored in ethanol for molecular analyses. The analyses of five microsatellite markers and partial mtDNA representing the hypervariable part of the control region (D-loop) were performed for each individual fish following Papoušek *et al*. [19]. Microsatellite loci GF29, MFW2, MFW7, J1 and J62 were amplified with previously published primers (see [19,20]). Microsatellite and mitochondrial genotypes in *C. carpio*, *C. gibelio* and their respective hybrids are shown in Table 1. The genetic differences in multilocus genotypes among two parental species and their respective hybrids were analyzed using principal coordinate analysis (PCoA) performed in GenAlEx 6.41 [21]. PCoA was performed using standardized genetic distances.

**Table 1:** **Microsatellite and mitochondrial genotypes in *C. carpio*, *C. gibelio *and their respective hybrids**

**Fish**	**GF29**	**MFW2**	**MFW7**	**J1**	**J62**	**Haplotype**
Hybrid 1	197/**257**	157/**253**	179/**188**	**123**/151	**158**/178	*C. gibelio*
Hybrid 2	197/**251**	157/**239**	175/**258**	**123**/147	**156**/178	*C. gibelio*
Hybrid 3	197/**273**	157/**213**	175/**258**	**125**/141	***148***/176	*C. gibelio*
Hybrid 4	203/**273**	157/**247**	179/**258**	**125**/157	**158**/178	*C. gibelio*
Hybrid 5	197/**255**	157/**237**	179/**258**	**123**/141	***148***/178	*C. gibelio*
Hybrid 6	193/**255**	157/**265**	179/**256**	**125**/159	**156**/176	*C. gibelio*
Hybrid 7	197/**255**	157/**239**	179/**258**	**127**/157	**162**/178	*C. gibelio*
Hybrid 8	197/**269**	157/**253**	179/**256**	**123**/157	**160**/176	*C. gibelio*
Hybrid 9	199/**251**	157/**249**	169/**196**	**123**/159	**156**/178	*C. gibelio*
Hybrid 10	197/**255**	157/**249**	175/**196**	**127**/151	**160**/178	*C. gibelio*
Hybrid 11	199/**255**	157/**249**	153/**196**	**123**/151	**156**/178	*C. gibelio*
Hybrid 12	197/**255**	157/**247**	179/**192**	**123**/151	**156**/178	*C. gibelio*
Hybrid 13	197/**255**	157/**245**	173/**242**	**125**/151	**156**/180	*C. gibelio*
*C. carpio*	**251-319**	**181-267**	**192-258**	**123-127**	**154-160**	*C. carpio*
*C. gibelio*	193-201	157	153-181	141-177	176-180	*C. gibelio*
*C. carpio* *	243-283	169-271	188-276	-	-	-
*C. gibelio* *	193-207	157	175-179	-	-	-
*C. gibelio* **	195-201	157	175-195	-	-	-

The allele size ranges according to Hänfling *et al*. [18] (shown with *) and Papoušek *et al*. [19] (shown with **) are included. Microsatellite alleles with presumed origin in *C. carpio* are shown in bold. The alleles outside the ranges of *C. carpio* or *C. gibelio* analyzed in this study are shown in italic.

Immediately after capture, the fish were transported live to the laboratory in barrels with the original oxygenated water and dissected within 24 hours. All fish were killed in the laboratory by severing the spinal cord. Standard length (in millimeters) and body weight (in grams) were recorded. Complete dissection of the fish was performed following the method of Ergens and Lom [22]. Fish were examined for all metazoan parasites following the standard techniques used in fish parasitology. All parasites were removed and fixed according to standard methods and then identified to species level using a light microscope (Olympus BX50) equipped with phase-contrast, differential interference contrast (DIC) and Digital Image Analysis (Olympus MicroImage™ for Windows 95/98/NT 7.0 (Olympus Optical Co.)). ANOVA followed by multiple Tukey post hoc tests was used to test the differences in fish body length between groups (*C. carpio*, *C. gibelio* and hybrids). The host range (i.e. host specificity in Table 2) was primarily identified using Moravec [23] and Pugachev *et al*. [24]. For *Paradilepis scolecina*, the host range was completed using Scholz *et al*. [25]. Even if the internet sources NHM Parasite-Host Database ( http://www.nhm.ac.uk/research-curation/scientific-resources/taxonomy-systematics/host-parasites/database/index.jsp) and Gyrobase (http://www.gyrodb.net) suggest wider host range for several parasite species, we found the same host species as indicated by the above references after filtering the host species representing the accidental findings, questionable host records, host species found only in captivity, parasite misidentification or doubtful identification. In addition, we removed the host species restricted to Asia (especially to Southeast Asia and Baltic Sea) because our study was performed at the local level. We also highlight that the host specificity shown for each parasite species in Table 2 corresponds to the records of the most frequent host species included in NHM Parasite-Host Database (i.e. *C. carpio*, *C. gibelio* and *C. carassius* were the most frequently recognized host species for the parasite species exhibiting a certain degree of host specificity). For delimitation host specificity, we applied the semiquantitative classification developed for congeneric monogeneans by Desdevises *et al*. [4]: strict specialist living on a single host species, intermediate specialist living on congeneric host species (i.e. *Carassius gibelio* and *Carassius carassius* in our study), intermediate generalist living on non-congeneric host species forming a monophyletic group (i.e. *Cyprinus carpio*, *Carassius gibelio* and *Carassius carassius* in our study), generalist living on phylogenetically distant host species (i.e. parasitizing fish species of different subfamilies within Cyprinidae or different cyprinid and non-cyprinid fish species). This classification essentially combined the information from basic and phylogenetic host specificity (as defined by Poulin *et al*. [2]).

**Table 2:** **The presence of metazoan parasite species in *C. gibelio*, *C. carpio *and their respective hybrids**

**Parasite species**	**Host specificity**	**CaGi**	**CyCa**	**Hybrids**
Monogenea				
*Dactylogyrus achmerovi*	strict specialist (CyCa)	-	100% (1–39)	15.4% (1–2)
*Dactylogyrus anchoratus*	intermediate generalist	85.7% (1–19)	16.7% (1–2)	46.2% (1–10)
*Dactylogyrus dulkeiti*	intermediate specialist	-	-	7.7% (1)
*Dactylogyrus extensus*	strict specialist (CyCa)	-	83.3% (1–13)	15.4% (1–2)
*Dactylogyrus formosus*	intermediate specialist	7.1% (2)	-	7.7% (1)
*Dactylogyrus molnari*	strict specialist (CyCa)	-	100% (5–21)	30.8% (1–4)
*Dactylogyrus intermedius*	intermediate specialist	28.6% (1–4)	-	-
*Dactylogyrus vastator*	intermediate generalist	14.3% (2–4)	-	15.4% (1–2)
*Dactylogyrus* spp. larvae	not evaluated	50% (1–8)	58.3% (1–9)	46.2% (1–6)
*Gyrodactylus katharineri*	generalist	7.1% (1)	-	-
*Gyrodactylus longoacuminatus*	intermediate generalist	35.7% (3–84)	8.33% (7)	53.8% (1–10)
*Gyrodactylus medius*	intermediate generalist	7.1% (1)	16.7% (2–6)	7.7% (1)
*Gyrodactylus shulmani*	intermediate generalist	21.4% (9–118)	16.7% (2–6)	15.4% (1–4)
*Gyrodactylus sprostonae*	intermediate generalist	100% (2–4754)	100% (3–500)	92.3% (1–1017)
*Gyrodactylus vimbi*	generalist	7.1% (2)	-	-
*Eudiplozoon nipponicum*	strict specialist (CyCa)	-	58.3% (1–7)	23.1% (1–3)
Crustacea				
*Argulus foliaceus*	generalist	21.4% (1–2)	75% (1–9)	46.2% (1–2)
Hirudinea				
*Piscicola geometra*	generalist	-	-	7.7% (1)
Digenea				
*Diplostomum spathaceum* larvae	generalist	21.4% (1)	58.3% (2–48)	69.2% (1–9)
Echinostomatidae fam.sp. larvae	not evaluated	-	-	7.7% (1–2)
*Paryphostomum radiatum* larvae	generalist	7.1% (1)	-	-
Cestoda				
*Khawia sinensis*	strict specialist (CyCa)	-	25% (1–5)	15.4% (1–2)
*Paradilepis scolecina*	generalist	50% (1–7)	-	7.7% (1–10)
Nematoda				
Nematoda sp.	not evaluated	7.1% (1)	-	7.7% (1–2)

The prevalence and intensity of infection (min-max) are included. CaGi – *Carassius gibelio*, CyCa – *Cyprinus carpio*.

ANCOVA followed by multiple Tukey post hoc tests were applied to test the differences in parasite abundance between fish groups (*C. carpio*, *C. gibelio* and hybrids); standard fish body length was used as covariate. Parasite abundance was log-transformed prior to ANCOVA. KW ANOVA followed by multiple comparison tests were used to compare the differences in abundance of two generalist parasite species between fish groups because in this case the distribution of parasite abundance did not fit a normal distribution. All analyses were performed using Statistica 10.0 for Windows, StatSoft Inc. The similarity between parasite communities was calculated using the qualitative Jaccard index on the parasite presence/absence data. The Jaccard index ranges from zero (no species is common between two host groups) to one (two host groups share the same parasite species).

This study was approved by animal care and use committee in Faculty of Science, Masaryk University in Brno (Czech Republic).

## Results

**Figure 1:**
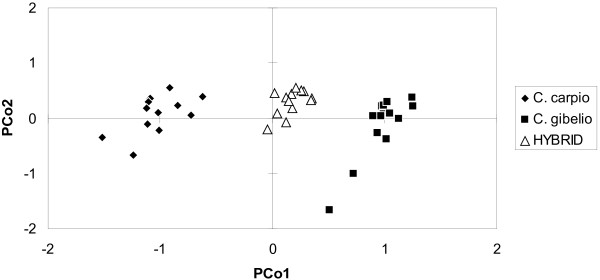
**The genetic differentiation of multilocus genotypes among *C. carpio*, *C. gibelio *and their respective hybrids based on principal coordinate analysis.**

All investigated specimens within each host group had a similar body size (standard length 240.07±13.24 mm for *C. gibelio*, 323.08±31.23 mm for *C. carpio* and 288±32.47 mm for their respective hybrids). ANOVA revealed a significant effect of host on standard body length (F_2,36_ = 30.75, p < 0.001). The standard body length of *C. carpio* was greater than that of *C. gibelio* (p < 0.001). The standard body length of hybrids was intermediate between *C. gibelio* (p < 0.001) and *C. carpio* (p = 0.013). The analyses of mtDNA showed that all hybrid specimens analyzed in this study possessed the haplotype of *C. gibelio* (Table 1), which indicated that the hybrids were descended from *C. gibelio* maternally. Microsatellite analysis of 13 putative hybrid specimens revealed the presence of nine alleles in the GF29 locus, nine alleles in the MFW2 locus, 11 alleles in the MFW7 locus, eight alleles in the J1 locus, and eight alleles in the J62 locus. For each of the loci GF29, MFW2, MFW7 and J1, one allele corresponded to *C. carpio* and the other to *C. gibelio* (based either on alleles actually observed or on size ranges described in Hänfling *et al*. [18] or Papoušek *et al*. [19] for all putative hybrids) (Table 1). With respect to locus J62, the alleles in two hybrid specimens (Hybrids 3 and 5) with a presumed origin from *C. carpio* were slightly outside the observed size range. The PCoA plot visualized the genetic differentiation between *C. carpio*, *C. gibelio* and the hybrids (Figure 1). The first two axes explained 63.57% of total variability.

A total of 23 metazoan parasite species were found in a whole fish sample including 15 species of Monogenea, 1 species of Crustacea, 1 species of Hirudinea, 3 species of Digenea, 2 species of Cestoda and 1 species of Nematoda (Table 2). Hybrids harbor more different parasite species than each of the parental species i.e. a total of 19 parasite species were found in hybrids whilst *C. carpio* harbor 12 and *C. gibelio* 15 parasite species. The similarity in parasite component communities between hybrids and parental species, based on the presence-absence of parasite species, was higher between hybrids and *C. carpio* (0.632) than between hybrids and *C. gibelio* (0.478). The similarity in parasite communities between *C. carpio* and *C. gibelio* was low (0.35).

The host specificity of each parasite species found on fish in our study is included in Table 2. Following the published data, 14 from 23 parasite species showed a certain degree of host specificity (using the semiquantitative classification of Desdevises *et al*. [4] for delimitation of host specificity). We identified strict specialists parasitizing solely *C. carpio*; intermediate specialists parasitizing *C. gibelio* and *C. carassius*; intermediate generalists parasitizing *Carassius* species (*C. gibelio* and/or *C. carassius*) and *C. carpio*. 13 from 14 parasites exhibiting a certain degree of host specificity measured in the phylogenetic context belong to Monogenea and one to Cestoda. Four monogenean parasites (3 *Dactylogyrus* species and *Eudiplozoon nipponicum*) and the cestode parasite *Khawia sinensis* were strictly host specific for *C. carpio*. In our fish sample, *Dactylogyrus* and *Gyrodactylus* parasites represented the genera with the highest species richness. All *Dactylogyrus* species and four *Gyrodactylus* species exhibited a certain degree of host specificity on the basis of above criteria (Table 2). Each parasite exhibiting a certain degree of host specificity (except for *D. intermedius*) was recorded in hybrids.

**Figure 2:**
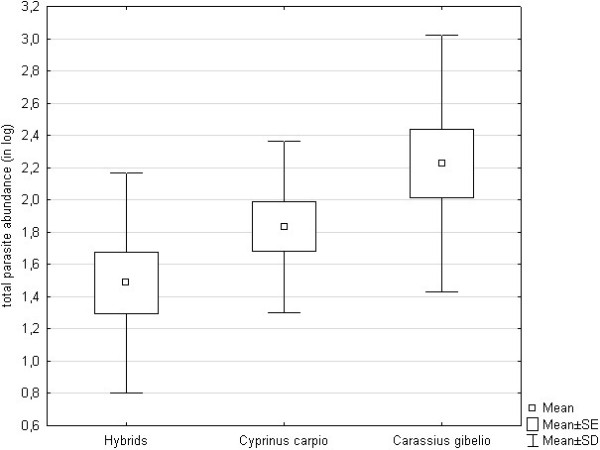
**Parasitism in *C. carpio*, *C. gibelio *and their hybrids measured by total parasite abundance.**

A significant effect of host on total parasite abundance was found (ANCOVA, whole model F_3,35_ = 3.60, p = 0.023 with standard length F = 2.61, p = 0.115 and host F = 5.28, p = 0.010). The total parasite abundance in hybrids tended to be lower when compared to both parental species (Figure 2). Multiple Tukey post hoc tests revealed a significant difference in parasite abundance between hybrids and *C. gibelio* (p = 0.019). However, no significant difference in total parasite abundance between hybrids and *C. carpio* (p = 0.409), or between *C. gibelio* and *C. carpio* (p = 0.311) was found.

**Figure 3:**
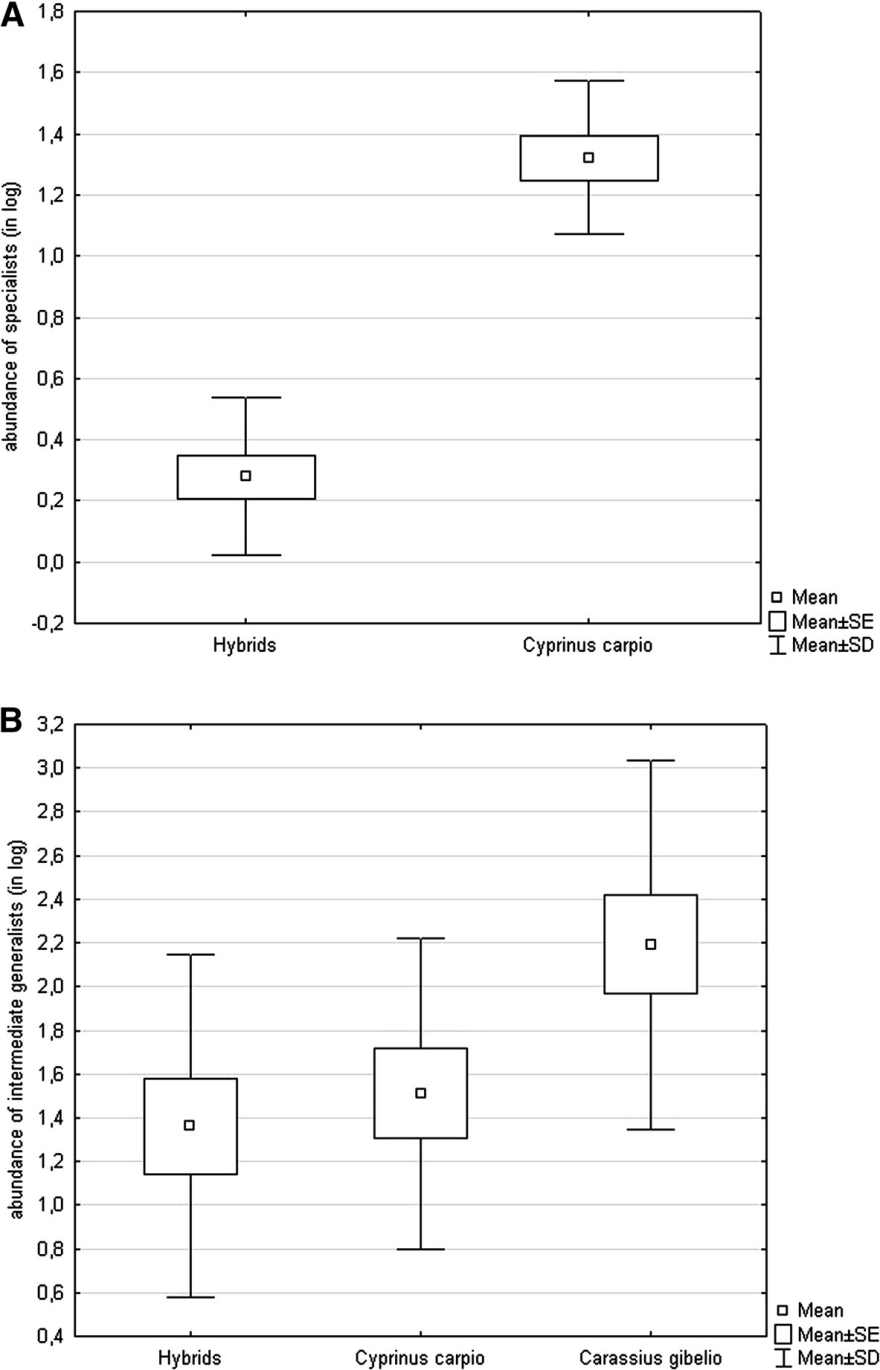
**The effect of interspecies hybridization on host specificity for (A) strict specialists in *C. carpio *and hybrids of *C. carpio *and *C. gibelio*, (B) intermediate generalists in *C. carpio*, *C. gibelio *and their hybrids.**

The prevalence and intensity of infection of each *Cyprinus carpio* specific parasite species (i.e. *Dactylogyrus achmerowi, D. extensus, D. molnari, Eudiplozoon nipponicum* and *Khawia sinensis*) were higher in *C. carpio* than in hybrids (Table 2). ANCOVA revealed a significant effect of host on the abundance of strict specialists (whole model F_2,22_ = 59.57, p < 0.001, body length F = 3.32, p = 0.082, host F = 73.14, p < 0.001) with *C. carpio* being significantly more parasitized than hybrids (Figure 3A). ANCOVA using only strictly specific monogenean parasites (i.e. after removing *Khawia sinensis*) revealed the same result (whole model F_2,22_ = 54.70, p < 0.001, body length F = 2.23, p = 0.150, host F = 69.77, p < 0.001). The prevalence and intensity of infection of intermediate specialists (i.e. the parasite species potentially infecting congeneric hosts - *C. gibelio* and *C. carassius*) were low in *C. gibelio* and hybrids (Table 2) and, moreover, *D. intermedius* was present solely in *C. gibelio*. Concerning intermediate generalists (i.e. *D. anchoratus*, *D. vastator*, *G. longoacuminatus, G. medius, G. shulmani* and *G. sprostonae*), they preferentially infected *C. gibelio* compared with *C. carpio* based on prevalence and intensity infection values (Table 2). The only exception from this pattern was *G. medius*, which is however considered as host specific for *C. carpio* by Moravec [23] and Pugachev *et al*. [24]. Considering intermediate generalists exhibiting the specificity for phylogenetically closely related hosts, ANCOVA revealed the significant effect of host on the parasite abundance (whole model F_3,35_ = 3.59, p = 0.023, body length F = 1.93, p = 0.174, host F = 4.71, p = 0.015). Tukey post hoc test revealed the significantly higher abundance of intermediate generalists in *C. gibelio* when compared to hybrids (p = 0.023) and there was also the trend for higher abundance in *C. gibelio* when compared to *C. carpio* (even if the difference was not statistically significant as revealed by p = 0.079) (see Figure 3B). The same results were found when removing *G. medius* from the analyses.

Concerning generalist parasites, the majority of them achieved low prevalence and intensity of infection values (Table 2). Such generalists were rarely found in *C. gibelio* and/or in hybrids. On the other hand, two generalist parasites *Argulus foliaceus* and *Diplostomum spathaceum* were present in *C. carpio*, *C. gibelio* and their hybrids. The prevalence of *A. foliaceus* was higher in *C. carpio* when compared to prevalence of this parasite species in *C. gibelio* and hybrids, whilst the prevalence of *D. spathaceum* was higher in hybrids when compared to the prevalence of this species in *C. carpio* and *C. gibelio* (Table 2). However, a significant difference in the abundance of these two parasite species was found only between parental species (for *A. foliaceus* KW ANOVA H_2,39_ = 9.02, p = 0.011, multiple comparison p = 0.019 and for *D. spathaceum* KW ANOVA H_2,39_ = 8.61, p = 0.014, multiple comparison p = 0.033) with *C. carpio* being more parasitized than *C. gibelio*.

## Discussion

The two fish species investigated in the present study, *C. gibelio* and *C. carpio*, are phylogenetically related cyprinids, probably of the same origin and historical dispersion [26]. In nature, they live in sympatry, which can promote their hybridization. The hybrids of *C. gibelio* and *C. carpio* analyzed in our study were confirmed using both meristic traits and molecular identification. All of the hybrid specimens bore the haplotype of *C. gibelio*, according to the analysis of mitochondrial DNA, and all of them expressed one allele of *C. gibelio* and one allele of *C. carpio*, according to microsatellite analysis. Thus, this suggests that all of them are F1 offspring of female *C. gibelio* and male *C. carpio*. Moreover, all of them express the same morphology.

Two principal outcomes result from our study. The first one indicates that hybrids are able to harbor more different species of parasites than each of their parental species. The majority of parasite species belong to Monogenea with *Dactylogyrus* and *Gyrodactylus* representing a large part of parasite diversity. However, the total abundance of parasites tended to be lower in hybrids when compared to *C. carpio* and *C. gibelio*. Thus, the interspecies hybrids probably have a lower susceptibility to at least some metazoan parasites when compared to their parental species, leading to low total parasite abundance in hybrids. A similar observation of low parasite abundance in interspecies fish hybrids was reported in an experimental study of infection by two *Gyrodactylus* species, *G. salaris* and *G. derjavini*, in pure-bred *Salmo salar* and *Salmo trutta* and their half-sib hybrids [17]. The study suggests that hybrids in nature may act as a reservoir for gyrodactylids, potentially supporting a wider diversity of species than their parental species and disseminating gyrodactylids of both host species. Our findings support this hypothesis as we showed such a pattern of infection for many monogenean species and several endoparasite species.

Contrary to our findings, a study of hybridization between two cyprinid species, *Alburnus alburnus* and *Rutilus rubilio* from Lake Mikri Prespa, Northern Greece, showed that the hybrids were highly susceptible to metazoan parasite infection including *Dactylogyrus* and *Diplozoon* (Monogenea), *Bolbophorus confusus* (larval stages of Digenea) and *Pomphorhynchus bosniacus* (Acanthocephala) [9]. This was explained by the different spatial and trophic position of hybrids, making them more exposed to parasites. Alternatively, it was proposed that hybrids could contain attractive substances from each of their parental species in their mucus or that perhaps immune defense expressed in parental mucus is impaired in hybrids [9]. The cyprinid species *C. gibelio* and *C. carpio* investigated in our study have the same spatial and trophic position, but their mucus substances or other immune mechanisms specific to the parental species might possibly represent potential mechanisms explaining the low abundance of parasites in *C. gibelio* x *C. carpio* hybrids. The studies performed with hybrid mice also show findings similar to those concerning fish hybrids described by Dupont and Crivelli [9], i.e. natural hybrids of mice are more infested by helminth parasites than both parental taxa [11,12], suggesting that the co-adapted gene systems controlling parasite infection are broken down in hybrid genomes because of high gene introgression.

The next outcome of our study was that hybridization between two phylogenetically closely-related non-congeneric cyprinid species affects the host specificity of metazoan parasites. Each of the two cyprinid species analyzed harbor specific parasite fauna, and especially specific monogenean species. *C. carpio* harbor strictly specific parasites, whilst *C. gibelio* harbor intermediate specialists, i.e. parasites that were previously recognized as specific to the congeneric hosts *C. gibelio* and *C. carassius*. In addition, these cyprinid species harbor the group of parasites exhibiting wider host specificity measured in phylogenetic context. The host range of such intermediate generalists was limited to phylogenetically closely-related *C. carpio*, *C. gibelio* and *C. carassius*. Concerning the host specificity of the most diverse parasite genera in *C. carpio* and *C. gibelio*, oviparous gill monogeneans of *Dactylogyrus* showed a high level of host specificity, i.e. each *Dactylogyrus* species was either strictly host specific or showed specificity for congeneric or non-congeneric phylogenetically related cyprinid species (as was shown for a large sample of *Dactylogyrus* species parasitizing cyprinid fish by Šimková *et al*. [5]). On the other hand, viviparous gill and skin monogenean parasites of *Gyrodactylus* showed low level of host specificity, i.e. the majority of *Gyrodactylus* species were intermediate generalists, and two of them were generalists infecting phylogenetically distant fish species. We showed that hybrids harbor the parasites exhibiting a certain level of host specificity.

Our study also shows that parasite life strategy, i.e. ectoparasitism versus endoparasitism, is not a feature limiting the effect of interspecies hybridization on host specificity, because *Khawia sinensis*, a cestode species, which is on the basis of published helminthological collections from the field studies (see review of Moravec [23]) considered as host specific to *C. carpio*, was also found in hybrids. However, the occurrence of this common carp-specific endoparasite in hybrid specimens cannot be explained by different feeding habitats between *C. gibelio* and *C. carpio*, as suggested by Dupont and Crivelli [9] (see above). It is more likely that the hybrids acquired some immune mechanisms specific to each pure host species, which facilitated the presence of both specific ecto- and endoparasites; however, because of co-adaptation, which is expected between the pure host genotype and specific parasite genotype, infection by specific parasites in interspecies hybrids is low. Our study showed that the abundance of all strictly specific common carp parasites was lower in hybrids than in pure species. In the case of intermediate generalists exhibiting phylogenetic specificity, both parental species *C. gibelio* and *C. carpio*, as well as their hybrids, were susceptible to infection and a significant effect of host on the parasite abundance of intermediate generalists was found when taking host body size into account. We showed that the prevalence and maximum intensity of infection of such parasites were higher in *C. gibelio* than in *C. carpio*, which indicates that *C. gibelio* could be considered as their preferred host (Šimková *et al*. [5] suggested that even a generalist parasite may prefer some host species within its host range) The infection by intermediate generalists in hybrids was lower when compared to the preferred *C. gibelio* or some intermediate generalists even tended to reach the values intermediate between parental species. Thus, host susceptibility traits for specific parasites are probably genetically controlled and a low susceptibility can be transferred as a dominant trait through interspecific crosses between different cyprinid species. However, such a hypothesis needs to be tested in the future. We cannot support strict co-adaptation between the host genotype of a pure species and a specific parasite genotype potentially explaining narrow host specificity. On the other hand, the hybrids investigated in our study seem to bear “favorable” recombinant genotypes associated with a low level of infection by specific parasites but facilitating their transmission. A similar finding, i.e. low infection by metazoan parasites (especially Monogenea), was found for hybrids of *P. toxostoma* and *C. nasus* from the Durance and Ardeche (South France) sympatric zones [10]. However, in their study, both the *P. toxostoma* genotype and the recombinant genotypes of hybrids were less susceptible to *Dactylogyrus* parasites when compared to the pure *C. nasus* genotype, suggesting co-evolutionary interactions between *C. nasus* and their *Dactylogyrus* species.

Dupont and Crivelli [9] showed that interspecies cyprinid hybrids from Lake Mikri Prespa harbor all *Dactylogyrus* host specific to the parental species and also that *Dactylogyrus* parasitizing species other than parental species were present in hybrids at low intensity. However, in their study, the pattern of high parasite infection by dominant host-specific *Dactylogyrus* was similar in the hybrids and pure species. This discrepancy between different hybridizing cyprinid systems could imply a different level of gene introgression (high gene introgression might induce the break-down of a co-adaptation gene system leading to high parasite infection in hybrids [12]), or it might be related to the different frequencies of hybrids in two systems (i.e. rare favorable *C. gibelio x C. carpio* hybrid genotypes versus frequent unfavorable *R. rubilio* and *A. alburnus* hybrid genotypes). Therefore, further ecological and genetic studies could be helpful in interpreting the pattern of parasite infection in hybridizing fish species.

Our study showed that even the infection by several generalist parasites found in all fish groups – *C. carpio*, *C. gibelio* and their hybrids – was shifted toward one parental species (i.e. the crustacean parasite *Argulus foliaceus* and the digenean parasite *Diplostomum spathaceum* preferentially infected *C. carpio*). This may suggest the function of some host species-specific immune mechanisms regulating also the infection of generalist parasites between different cyprinid species.

## Conclusions

The interspecies hybridization of two non-congeneric phylogenetically closely-related cyprinid species *C. carpio* and *C. gibelio* affects the host specificity of their metazoan parasite species, i.e. the parasites exhibiting a certain degree of host specificity occur in hybrid specimens. Strict specialists showed a preference for parental species when compared with hybrids, likely resulting from the co-adaptation of host and parasite genotypes. However, on the basis of prevalence and maximum intensity of infection, the majority of intermediate generalists exhibited a preference for *C. gibelio* (probably also determined genetically) when compared to *C. carpio*, likely resulting in intermediate infection values for some parasite species in recombinant genotypes of F1 hybrids compared to parental host genotypes or low infection values for other parasite species found in both hybrids and *C. carpio*.

## Competing interests

The authors declare that they have no competing interests.

## Authors’ contributions

AŠ designed the study and drafted the manuscript. AŠ, MD and LV participated in the field studies to acquire fish and parasite data. MD determined the parasites. IP carried out the molecular analyses and statistical analysis of molecular data. AŠ analyzed the whole data. AŠ, MD, LV and IP were involved in drafting the manuscript or revising it critically for important intellectual content. All authors read and approved the final version of the manuscript.
